# Low humoral responses to human cytomegalovirus is associated with immunological treatment failure among HIV infected patients on highly active antiretroviral therapy

**DOI:** 10.11604/pamj.2017.28.131.10480

**Published:** 2017-10-10

**Authors:** Mariam Mwinjuma Mirambo, Ndealilia Senyaeli, Stephen Eliatosha Mshana

**Affiliations:** 1 Department of Microbiology and Immunology, Weill Bugando School of Medicine, P.O. Box 1464, Mwanza, Tanzania

**Keywords:** IgG, HCMV, HIV, immunological failure

## Abstract

Human cytomegalovirus (HCMV) is one of the opportunistic infections associated with significant morbidity and mortality among HIV/AIDS patients especially before introduction of antiretroviral therapy (ART). Little is known regarding the humoral immune response against HCMV in relation to CD4 counts among HIV infected individuals. A total of 90 achieved sera from HIV infected patients attending Bugando Medical centre care and treatment centre (CTC) aged 18 years and above were retrieved and analyzed. Sociodemographic data were collected using structured data collection tool. Detection of specific HCMV antibodies was done using Indirect Enzyme Linked Immunosorbent Assay (ELISA). Data were analyzed by using STATA version 11. A total of 90 HIV infected patients were enrolled in the study whereby 36(40%) had immunological treatment failure. The mean age of the study participants was 39±12.3 years. The Prevalence of specific HCMV IgG antibodies was 84(93.3%, 95% CI: 88-98.5) while the prevalence of specific HCMV IgM antibodies was 2(2.3% 95% CI: 0.8-5.4). The median CD4 counts at 6 months and 12 months on HAART were significantly high in treatment success group. At 12 months of HAART as CD4 counts increases the HCMV IgG index value was also found to increase significantly, p=0.04. Significant proportion of HIV infected individuals was infected with HCMV. Higher median HCMV IgG titers were observed among patients with immunological treatment success. There is a need to investigate humoral immune responses in HIV infected individuals in relation to CD4 counts against various infectious diseases in developing countries where most of these infections are endemic.

## Brief

Human cytomegalovirus (HCMV) has been known to establish latency following primary subclinical infections in immunocompetent individuals [1, 2]. In these individuals reactivation may occur periodically without any observed sequels. However, in deficiency of host-derived antiviral immune responses reactivation often cause pathological changes that may results into invasive disease [3-5]. Long-lasting immunity in response to HCMV primary infection serves to control subsequent HCMV reactivation in the host which is essential for preventing uncontrolled viral replication and serious HCMV disease [6]. In this context humoral and cell mediated immunity plays a crucial role in sustaining the balance between maintenance and reactivation which may results into pathological changes among these patients in case of any disruption. Human immunodeficiency virus (HIV) infected patients can control this balance when they start therapy as the CD4 counts and other immunological markers increases. However, in patients with immunological treatment failure it has been hypothesized that low CD4 counts may indicate failure of immune system to mount immunological responses to various antigens including latent HCMV or new infections [7-9]. Whether immunological treatment failure can affect generation of antibody responses to HCMV in these patients or not has never been established. This study investigated the specific HCMV IgG titers values in relation to CD4 counts as a marker of immunological treatment failure among HIV patients on highly active antiretroviral therapy (HAART).

A total of 90 achieved sera from HIV infected patients attending Bugando Medical centre CTC aged above 18 years were retrieved and analyzed. The sera were obtained at 12 months of HAART. Sociodemographic and other relevant information were obtained from patients' records. Detection of specific HCMV IgG antibodies was done using commercial indirect enzyme-linked immunosorbent assay (PishtazTeb, Tehran, Iran) according to manufacturer's instructions [10-13]. Data analysis was done by using STATA version 11. Proportions and confidence intervals were determined. Two-sample Wilcoxon rank-sum (Mann-Whitney) test was done to compare median CD4 counts among participants with immunological treatment failure and those with immunological treatment success. Scatter diagrams were drawn using patients CD4 counts records at baseline, at 6 months and 12months in relation to HCMV IgG index values. Regression analysis was done to determine the statistical significance of the correlation. The protocol to conduct the study obtained waiver of consent from the Joint Catholic University of Health and Allied Sciences/Bugando Medical Centre (CUHAS/BMC) research ethics and review committee with clearance number CREC.208/2016. Of 90 HIV infected patient's sera, 36(40%) were from patients who had immunological treatment failure. The mean age of the study participants was 39±12.3 years. Majority of participants were male 61(67.8%) and those with primary education were 63(70%). Regarding marital status, unmarried individuals formed majority 61(67.8%) of the study participants. In addition 54(59.9%) of the participants were employed.

**Figure 1:**
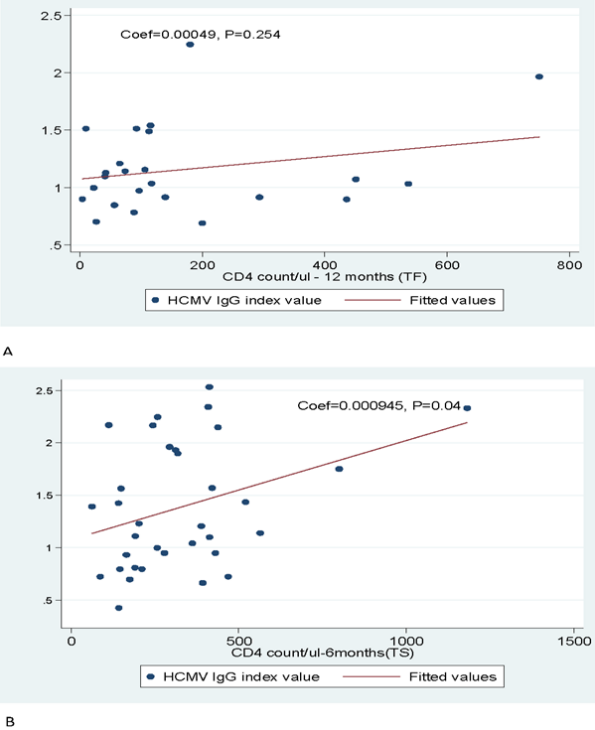
HCMV index value of HIV infected individuals in relation to CD4 counts at baseline, 6 months, 12 months. TF: Treatment Failure, TS: Treatment Success

A total of 84(93.3%, 95% CI: 88-98.5) sera had specific HCMV IgG antibodies while only 2(2.3% 95% CI: 0.8-5.4) had specific HCMV IgM antibodies. Higher rates of HCMV IgG seroprevalence have been similarly observed elsewhere among HIV infected individuals [14, 15]. In comparison to previous studies the reported prevalence of HCMV IgM antibodies was lower [16]. As per WHO citeria [17], the median CD4 counts were significantly higher among patients who had immunological treatment success at 6 months and 12 months (Table 1). On the regression analysis there was no significant correlation between baseline CD4 counts and HCMV IgG index value (p=0.45). This could be due to sigfnificant low CD4 counts which might have contributed to the impared function of CD4 due to high viral load [17, 18]. Correlation with bordeline significant between CD4 counts and HCMV IgG index values was oberved at 6 months on HAART while significant correlation between CD4 counts and HCMV IgG index value was observed at 12 months on HAART, p=0.04, (Figure 1). Generally we observed that at 6 months and 12 months there was significant relation between IgG titers and CD4 counts in the treatment success group. This could be due to increase immunocompetency which is associated with HAART treatment. Previous study [19] showed that the increase in CD4 count and function is associated with increased humoral response. In addition no significant difference was observed on the HCMV antibody titers in relation to age in both groups, signifying that HIV and CD4 counts are only factors influencing HCMV humoral immune responses.

**Table 1:** Median CD4 counts in relation to treatment status of HCMV IgG positive

	Treatment failure		Treatment sucess		P
Interval	CD4 counts (IQR)	N	CD4 counts(IQR)	N	
Baseline	84(31-158)	34	104(41-178)	50	0.45
At 6 months	132(58.5-280.5)	32	229(132-229)	50	0.01
At 12 months	101.5(49.5-190)	24	311(175-414)	34	<0.01

In conclusion significant proportion of HIV infected individuals were HCMV IgG sero-positive and the HCMV IgG titers significantly increased as CD4 counts increases. Further studies to investigate the humoral immune response in HIV infected individuals in relation to CD4 counts against various infectious diseases are warranted in developing countries.

## Competing interests

The authors declares that they have no competing interests.
